# Qualitative research to inform hypothesis testing for fidelity-based sub-group analysis in clinical trials: lessons learnt from the process evaluation of a multifaceted podiatry intervention for falls prevention

**DOI:** 10.1186/s13063-020-04274-6

**Published:** 2020-04-21

**Authors:** Arabella Scantlebury, Sarah Cockayne, Caroline Fairhurst, Sara Rodgers, David Torgerson, Catherine Hewitt, Joy Adamson, on behalf of the REFORM study

**Affiliations:** York Trials Unit, Department of Health Sciences, University of York, York, YO10 5DD UK

**Keywords:** Process evaluation, Randomised controlled trials, Fidelity, Mixed methods, Falls, Elderly, Ageing, Qualitative

## Abstract

**Background:**

Ensuring fidelity to complex interventions is a challenge when conducting pragmatic randomised controlled trials. We explore fidelity through a qualitative process evaluation, which was conducted alongside a pragmatic, multicentre, two-arm cohort randomised controlled trial: the REFORM (Reducing Falls with Orthoses and a Multifaceted podiatry intervention) trial. The paper aims, through a qualitative process evaluation, to explore some of the factors that may have affected the delivery of the REFORM intervention and highlight how project-specific fidelity can be assessed using a truly mixed-methods approach when informed by qualitative insights.

**Design:**

Semi-structured qualitative interviews carried out as part of a process evaluation. Interviews were analysed thematically.

**Setting:**

Seven NHS trusts in the UK and a University podiatry school in Ireland. Interviews were undertaken face-to-face or over the telephone.

**Participants:**

Twenty-one REFORM trial participants and 14 podiatrists who delivered the REFORM intervention.

**Results:**

Factors affecting fidelity included: how similar the intervention was to routine practice; the challenges of delivering a multifaceted intervention to a heterogeneous older population; and practical issues with delivery such as time and training. Trial participants’ views of the intervention, whether falls prevention is a personal priority, their experience of being part of a trial and individual factors such as medical conditions may also have affected intervention fidelity.

**Conclusions:**

Our process evaluation highlighted factors that were perceived to have affected the fidelity of the REFORM intervention and in doing so demonstrates the importance of considering fidelity when designing and evaluating pragmatic trials. We propose a number of recommendations of how important project-specific insights from qualitative work can be incorporated into the design of fidelity measurement of future trials, which build on existing conceptual fidelity frameworks. In particular, we encourage adopting a mixed-methods approach whereby qualitative insights can be used to suggest ways to enhance quantitative data collection facilitating integration through hypothesis generation, hypothesis testing and seeking explanation for trial findings. This will provide a framework of enabling measures of fidelity to be incorporated into the understanding of trial results which has been relatively neglected by existing literature.

**Trial registration:**

ISRCTN Registry: ISRCTN68240461. Registered on 01/07/2011.

## Introduction

Pragmatic randomised controlled trials (RCTs) are often used to evaluate the effectiveness of complex interventions – those that include multiple interacting components [1, 2]. Intervention fidelity, the extent that interventions are implemented as intended [3], is a particular problem when designing and evaluating pragmatic RCTs [4]. Given that intervention fidelity is a potential moderator of the relationship between interventions and their intended outcomes, it should be a priority when designing and evaluating RCTs. However, measurement of fidelity has been piecemeal at best and is often overlooked.

One of the challenges researchers face when considering fidelity is the uncertainty as to how the concept should be defined and measured. There are numerous examples of reviews and primary research papers [5–9] that have aimed to define the key elements of fidelity, with the concept becoming more complex as research has increased [5]. In 2007, Caroll et al. undertook a critical review of existing research on conceptualising fidelity. The resulting theoretical framework considers fidelity to consist of seven elements: adherence to an intervention; exposure or dose; quality of delivery; participant responsiveness; programme differentiation; intervention complexity; and facilitation strategies [5]. This framework has since been updated by Hasson (7) to incorporate two additional moderating factors, namely context and recruitment (Table 1).

**Table 1:** The REFORM trial

Objectives:	To determine the clinical and cost-effectiveness of a multifaceted podiatry intervention for preventing falls in community dwelling older people at risk of falling, relative to usual care.
Design:	A pragmatic, multicentre RCT with an economic evaluation and qualitative study. 1010 participants aged ≥ 65 years were randomised (intervention, n = 493; usual care, n = 517) via a secure, remote randomisation service.
Interventions:	All participants received a falls prevention leaflet and routine care from their podiatrist and GP. The intervention also included: footwear advice; footwear provision (if required); foot orthoses; and foot and ankle strengthening exercises.
Control:	Participants in the control group continued to receive usual care from their podiatrist and GP, which m ay have included prescription of an orthosis and footwear advice. They also received the same falls prevention leaflet sent to the intervention participants.
Primary outcome:	The primary outcome was the incidence rate of falls per participant in the 12 months after randomisation.
Trial status:	Completed (ISRCTN68240461)
Funder:	NIHR Health Technology Assessment

*GP* general practitioner, *NIHR* National Institute for Health Research *RCT* randomised controlled trial

We adopt Hasson’s [7] definition of fidelity and explore fidelity of the REFORM – Reducing falls with orthoses and a multifaceted podiatry intervention trial. REFORM was a pragmatic, multicentre, two-arm RCT that aimed to determine the clinical and cost-effectiveness of a multifaceted podiatry intervention for preventing falls in community-dwelling older people at risk of falling, relative to routine podiatry care. The intervention was complex and consisted of: footwear advice (and footwear provision if required), an orthotic insole or review of an existing prescription; a programme of foot and ankle balance exercises; and a falls prevention leaflet. Further details of the REFORM trial are provided in Table 2 and the study has been published elsewhere [10, 11].

**Table 2:** Nine elements of intervention fidelity adapted from Hasson et al. [7]

Element of implementation fidelity	Description
Adherence	Whether an intervention is being delivered as intended
Exposure or dose	Whether the amount of an intervention received by participants (frequency and duration) is intended
Quality of delivery	The way that those responsible for delivering the intervention deliver it
Participant responsiveness	How far participants respond to, or are engaged by, an intervention
Program differentiation	Identifying unique features of different components of programs and identifying which elements are essential
Intervention complexity	Complexity of an idea can act as a barrier to adoption - how complex is the intervention?
Facilitation strategies	When aiming to evaluate implementation fidelity, what are the specific strategies put in place to support implementation, e.g. provision of manuals, training and incentives. How were these strategies perceived by those involved in delivery?
• Recruitment	The recruitment strategies used to attract individuals to the intervention – what are the challenges to involvement?
• Context	What factors at political, economic, organisational and work group levels affected implementation?

• Denotes new moderating factors for understanding fidelity

In the present paper, we present findings of a qualitative process evaluation that was conducted alongside the REFORM trial. Through interviews with podiatrists who delivered the REFORM intervention and trial participants, we aim to explore some of the factors which affected the fidelity of the REFORM intervention. We aim to demonstrate the pivotal role that qualitative research can play in the identification of a credible a priori hypothesis for fidelity-based compliance and sensitivity analysis in order to assist with the interpretation of the findings from clinical trials – an aspect of fidelity assessment that has previously received relatively little attention.

## Methods

A qualitative process evaluation using semi-structured interviews with podiatrists who delivered the REFORM intervention and trial participants within England and the Republic of Ireland was conducted. This research design enabled us to explore fidelity of the REFORM intervention and obtain insight into the challenges of ensuring intervention fidelity when undertaking pragmatic trials of complex interventions. Data collection took place between November 2013 and March 2016. NHS Research Ethics Committee and Galway REC approved the study on 9 November 2011 and 26 April 2011, respectively. The University of York, Department of Health Sciences Research Governance Committee approved the study on 2 August 2011. Research management and governance approval were obtained from individual NHS trusts.

### Sampling and recruitment

All REFORM trial participants living in the Yorkshire or Lincolnshire areas who indicated on the main trial consent form that they were willing to be contacted about associated REFORM studies and who had received the REFORM intervention were eligible for the qualitative study. A purposive sampling frame was used to recruit a heterogeneous sample of trial participants to ensure maximum variation according to age, gender and history of falls [12]. Participants were sent a letter that included a participant information sheet and consent form and explained that a qualitative researcher would contact them via telephone to discuss the qualitative study and, if the participant was willing, to arrange a time for the interview.

As podiatrists delivering the intervention were based in a wide variety of clinics, it was anticipated that their views and experiences of delivering routine podiatry services and the REFORM intervention would differ. For example, clinics were located in different geographical regions with some podiatrists working in biomechanics, while others worked in routine podiatry clinics. The Principal Investigator at each recruiting site, therefore, invited all of the 28 podiatrists who delivered the REFORM intervention to take part in the qualitative study. Podiatrists were asked to contact the research team directly if they wished to take part.

### Participants

Fifteen podiatrists and 21 participants from the REFORM trial were interviewed. The sample of REFORM trial participants included 10 men and 11 women aged 65–87 years. Fifteen trial participants said that they lived with their spouse and/or other family members and the remaining six lived alone. Participating podiatrists represented seven NHS trusts and a University podiatry school in Ireland, had 6–32 years of experience and represented various grades of podiatrist, including one at band 5, six at band 6, six at band 7 and two at band 8.

### Data collection

Interviews with trial participants were conducted face-to-face in participants’ homes or at the University of York and on average lasted 40 min. Podiatrist interviews lasted 30–70 min and were conducted via telephone or at the premises where podiatrists were based. All interviews were semi-structured and followed a topic guide (Additional files 1 and 2). During interviews, podiatrists were asked: how the REFORM intervention compared to routine practice; acceptability and barriers to implementation among service providers; and acceptability and adherence among service users. Trial participants were asked about their experiences of being part of a RCT, general understanding of improving balance and reducing falls, and views and experiences of the intervention. Written informed consent was taken from all participants before taking part in the study.

### Analysis

Interviews were audio-recorded and transcribed verbatim. To ensure that a systematic approach to analysis was adopted, interviews were analysed thematically, according to the six stages outlined by Braun and Clarke: familiarisation; generating initial codes; searching for themes; reviewing themes; defining and naming themes; and data reporting [13]. Theme and sub-theme development was initially largely deductive, using a priori codes dictated by the topic guide while allowing for emergent themes. After the initial thematic analysis (by authors SC and AS), which was undertaken for the main HTA report [11], a secondary analysis was conducted (AS) to explore the extent to which themes developed in the initial analysis related to fidelity. At all stages in the analysis, coding and interpretation were discussed within the qualitative team (SC, AS and JA). Suggestions for future statistical analysis and integrated mixed-methods analysis of qualitative and quantitative material relating to exploration of the impact of fidelity on trial findings were discussed with the quantitative team (CF, DT and CH).

A reflexive approach was taken to data analysis. Interviewers (AS, SC) were academic research fellows with no podiatry training. SC was the REFORM trial manager and AS had no prior knowledge or experience of podiatry interventions. JA is also an academic researcher with no previous knowledge or experience of podiatry care. The background of the qualitative research team placed them in a neutral position in relation to any prior expectations to the study intervention.

## Results

We discuss each theme and sub-theme in turn. To aid the interpretation of our findings and to place them in the context of the wider fidelity literature, we use Hasson’s framework for conceptualising fidelity in the discussion [7].

How does the REFORM intervention compare to routine practice?

Whether podiatrists prescribed any of the components of the REFORM intervention in their routine practice may have influenced their ability, or willingness, to deliver the intervention. With the exception of footwear provision, all other components of the intervention (orthoses, exercises and footwear advice) are to some extent provided routinely. However, the types of orthoses, the number and types of exercises, and level of footwear advice varied across podiatry services. Podiatrists described one of the biggest differences between the REFORM intervention and routine practice as being that during the trial, standardised exercises and orthoses were recommended to all participants, whereas in routine practice, different exercises and orthoses may be prescribed depending on the patient’s individual need and what is available at each organisation.

‘*I mean all the exercises are used, they’re just not used altogether and the difference with the REFORM trial was it gave a package of exercises rather than standalone exercises.*’ (Podiatrist)

While the majority of podiatrists spoke positively of the intervention and its components, these differences between routine practice and the REFORM trial led to some cause for concern among podiatrists. For example, podiatrists explained that they were reluctant to prescribe exercises and orthoses to participants without a full biomechanics assessment. The majority of podiatrists were willing to incorporate the REFORM intervention into routine practice, should it be proven to be effective. However, a number of concerns were raised about the feasibility and costs of delivering the intervention in its current state. For instance, during the trial, the time for podiatry appointments was increased from 30 min to 60 min to enable all elements of the intervention to be delivered. Podiatrists were also uncertain that their organisations would provide funding to allow footwear to be provided routinely. As a result, a number of podiatrists described how, since the trial, they have been incorporating some, but not all, elements of the REFORM intervention (e.g. the exercise booklet) into their practice.

‘*I don’t think so, I think the cost implications of doing that and you’re looking at a shoe costing between 60 and 75 pounds, it would be too much of a hit.*’ (Podiatrist)

### Patients’ views of the intervention

Whether participants felt that taking part in the REFORM trial would improve their balance and help to reduce their number of falls may have contributed to their willingness to adhere to the intervention. Although one participant reported not finding the study beneficial, others felt that being part of the study had increased their awareness of falls and their confidence when walking. This is also true for the individual intervention components, as patients’ perceptions of whether a certain element of the intervention was of benefit may have affected their adherence to it throughout the trial. For example, while some participants felt that the insoles and exercises had given them more support and had improved their confidence and balance, others perceived them to have made no difference and, in one case, to have had a detrimental effect on their feet. Some participants also discussed having previously received footwear advice, exercises and/or orthoses outside of the trial. These prior experiences, particularly if negative, influenced whether participants believed the REFORM intervention would have a positive effect on their balance and reduce their number of falls.

‘*I don’t know whether it’s because I’ve got them (the insoles) in and it gave me more confidence, but I don’t know, I could just tell there and then it made a difference to my walking.*’ (Trial participant)

### Challenges of delivering a multifaceted intervention to an elderly population

The extent that podiatrists considered the intervention to be of benefit to the target population may also have influenced how it was delivered. Podiatrists spoke positively of the intervention and felt that, for those for whom the intervention was appropriate, it would improve their balance and reduce falls. While considered appropriate for the majority of participants, some podiatrists felt that the intervention was better suited to patients who were elderly but still ‘fit, healthy and mobile’, and so could engage with all components of the intervention. Podiatrists were sceptical as to whether the intervention would be of benefit to patients who were very frail and/or who had significant medical and/or podiatry problems (e.g. arthritis). In particular, some of the exercises were considered unsuitable or too challenging for frail patients and so in some cases, some or all elements of the intervention were not prescribed. Despite most concerns relating to the suitability of the intervention to the frail elderly, there was also a minority of participants that were included in the trial that they felt did not have balance problems and so the intervention was considered unnecessary.

‘*It’s more suited to the patients for that category where they’re still fit and healthy and they’re able to mobilise quite well. The moment they become so frail that they’re at risk of falls anyway just from say, postural hypertension, etc. I don’t think the intervention is going to help them that much.*’ (Podiatrist)

Podiatrists’ concerns regarding the suitability of the intervention were echoed by a number of trial participants who described how their health or certain medical conditions prevented them from being able to adhere to, and in some cases undertake, certain elements of the intervention. For example, bunions prevented some participants wearing insoles. A number of participants also reported that their health deteriorated over the course of the trial affecting their ability to continue with the intervention.

‘*I’ve got a bad heart, it doesn’t take me long to get out of breath, so I found them hard work, very hard work. One I couldn’t do. The one where you had to stand up with your back against a wall, I couldn’t do that because I couldn’t balance on one leg because this knee is so bad.*’ (Trial participant)

Concerns about the appropriateness of the intervention for certain participants led to some podiatrists modifying or adapting elements of the intervention to accommodate the individual patient’s needs. In particular, podiatrists reported difficulties with prescribing the full package of exercises to an elderly population and so described how they reduced or modified the number and types of exercises that were prescribed.

‘*We asked them to modify their approach and to do as many of the exercises as they could do but not to feel too bad if they had to reduce the frequency of the exercise or maybe even miss one exercise out, for instance, if they had osteoarthritis of the first MTP, big toe joint, we would say to them, don’t worry about getting on tiptoe but do and try and do the other ones.*’ (Podiatrist)

### Falls prevention: a priority for the patient?

The extent to which falls prevention is a priority for participants is another factor that may have influenced whether they adhered to the REFORM intervention. In our sample, only a small proportion of interviewed participants reported having experienced falls that had led to significant injury (i.e. broken bones, hospitalisation), which for some translated into a ‘fear of falling’. However, the majority of participants reported being worried about general unsteadiness as opposed to having a fear of falling per se. Associated with this, whether an individual believes that they have a ‘problem’ with falls may also influence their motivation to adhere to the intervention. Despite a large proportion of interviewees reporting using walking aids (e.g. stair rails, walking sticks) and having experienced multiple falls, previous falls were attributed to external circumstances or one-off errors, such as ‘not paying attention’, wearing bad shoes, and poor surfaces and weather conditions, and did not necessarily translate into a perception that falls were a problem for them.

‘*I’m careful about it but I don’t think I worry too much about it. I mean, the sort of fear of falling wouldn’t stop me doing something I wanted to do. I am consciously careful where I am. I think nearly all the falls I’ve had have been sort of putting my foot onto something but it’s not there, it’s 2 inches lower that kind of thing. I had a fall in Switzerland, years ago now. It was in a public toilet and I negotiated the step into it and when I was coming out, I’d forgotten it was there and I’m walking along merrily and suddenly there was no step and I hurtled into the wall. I thought I’d detached my retina because the side was … I hadn’t. I’d just clipped my forehead and the blood was running down by my spectacles.*’ (Trial participant)

### Practical issues with adhering to and delivering the intervention

Podiatrists and trial participants also discussed a number of practical issues that affected their ability to deliver and undertake the REFORM intervention.

#### Training and support

The trial team provided training to all podiatrists involved in delivering the REFORM intervention. Podiatrists spoke positively about the training they received and considered it to have improved their understanding of, and confidence in, delivering the REFORM intervention as it was intended. Podiatrists praised the duration (half a day) and delivery (presentation and role play) of training and the trainer’s competence in communicating the various elements of the intervention. Podiatrists also spoke positively about the additional refresher training that they received and the availability of the research team who provided added support throughout the trial. However, a small number of podiatrists were critical of the timing of training. One podiatrist who was required to deliver the intervention immediately after training felt that more time was needed for them to ‘digest’ the information provided. In contrast, other podiatrists felt that receiving training for such a complex intervention too far in advance of their first trial patient was detrimental.

‘*I mean, [name of trainer] went through everything with us before we were let loose on the patients. She went through everything. All of us were there at the time and we had a chance to try and see how we actually did it and if we were having difficulties with anything, [name of trainer] would step in, you know, she seemed to know everything and everything was well demonstrated for us.*’ (Podiatrist)

#### Information overload?

Podiatrists and trial participants described how, as this was a multifaceted intervention, remembering how to deliver and undertake all elements of the intervention was a challenge. The additional resources (e.g. exercise booklets, DVDs) provided to podiatrists and trial participants were perceived to mitigate against this to some extent; however, provision of these resources to trial participants was patchy. Podiatrists also discussed the importance of ‘selling the intervention’ to participants to ensure that they understood the intervention and adhered to it as intended. Follow-up visits were also considered vital in ensuring that participants were adhering to the intervention, particularly given that the majority of the intervention was undertaken at participants’ homes. Some podiatrists therefore felt that given the complexity of the intervention and target population, additional follow-up visits would have been beneficial.

‘*I think it is very much about how the research is sold to the patient really. I think it’s quite intensive. I think there is a lot of information given to the patients in the first assessment, but I think that the advice leaflet and the DVDs probably helped to support that. I think there has been a couple of issues about patients coming back and not quite having understood what they are supposed to do.*’ (Podiatrist)

#### Time

For a number of podiatrists, finding the time to deliver and explain the various elements of the REFORM intervention, in addition to randomisation and trial paperwork, was challenging. While the majority of podiatrists felt that sufficient time had been allocated to allow for this during the trial, some podiatrists reported finding it difficult to ‘juggle the trial and routine patients’ and were uncertain about the feasibility of delivering the REFORM intervention routinely.

‘*Usually with a new patient, times are varied between 45 min and 50 min for a biomechanical assessment. We did find that with the trial that we did need almost that full hour because there’s a lot of explaining to do.*’ (Podiatrist)

#### Issues with delivering individual intervention components

A number of issues with delivering specific components of the REFORM intervention were discussed. For example, while the footwear assessment was considered straightforward, a number of podiatrists reported difficulties with the footwear sizing guide, particularly for participants with ‘complex feet’. Orthoses were generally considered easy to fit as the insole was slim and could be trimmed or modified by the podiatrist if necessary. However, there was some concern about the level of arch support it provided and the appropriateness of prescribing an orthotic without a full biomechanics assessment. Despite being fitted by the podiatrist, one of the main factors affecting whether participants continued to wear the trial footwear was comfort. Podiatrists explained how despite there being a selection of shoes available for participants to choose from, and the importance of ‘good footwear’ for falls prevention being explained to participants, some were reluctant to change their footwear for aesthetic reasons and continued to wear inappropriate shoes.

‘*It was a bit confusing with the sizing, that’s what I personally found anyway especially with the two different providers that we had.*’ (Podiatrist)

### Trial experience

Participants’ willingness to take part in research and their experiences of being part of the trial may also have influenced their adherence to the intervention. A number of participants explained that they had been willing to take part in the research and saw it as an opportunity to ‘give back’ for the care they have received over the years. Additionally, although a small number of participants viewed the trial paperwork (completing monthly falls calendars) to be a burden, and in some cases had forgotten to do so, others did not consider this to be a problem and viewed the trial as an interesting experience. Whether participants had taken part in research previously may also have affected their understanding of the research process and the importance of adhering to the intervention; previous experience of taking part in research was mixed in this sample.

‘*I’ve had some wonderful care and kindness from the hospital here in [place name]. So, any little thing that I could do to pay it back a little, I’m always quite happy to do it.*’ (Patient)

## Discussion

This qualitative process evaluation explored the fidelity of the REFORM intervention – a complex, multifaceted podiatry intervention that aimed to prevent falls in older people. During interviews, trial participants and podiatrists provided a number of examples of situations where the intervention was not delivered as intended and identified a number of factors that were perceived to have affected the fidelity of the REFORM intervention (Table 3).

**Table 3:** Relationship between qualitative process evaluation and mixed-methods fidelity analysis

Element of intervention fidelity	Qualitative theme(s)	Explanation	Informing design of data collection	Potential interface with quantitative analysis/interpretation of trial findings
*Content; Frequency/Duration* *(dose, dose delivery); Coverage (reach)* Was each of the intervention components implemented as intended? Is the frequency and duration of the intervention as intended? What proportion of the target group participated in the intervention?	Challenges of delivering a multifaceted intervention to an older (≥ 65 years) population	Podiatrists do not prescribe elements of the intervention for health or medical reasons Podiatrists modify and adapt intervention components to suit individuals’ needs and capabilities	Qualitative work to identify appropriate adaptations for trial population	Compliance analysis of trial results according to delivery of intervention as intended (including appropriate adaptations)
*Recruitment* What recruitment procedures were used? What factors affect attrition?	Challenges of delivering a multifaceted intervention to an older population	Podiatrist felt the intervention was better suited to older patients who were still ‘fit, healthy and mobile’	Question on ‘intervention log’ of perceived suitability of patient for intervention	Quantitative description of characteristics of perceived suitability for the intervention by service providers Subgroup analysis of trial results according to service provider rated suitability of the intervention for the participant
*Participant responsiveness* How far participants respond to, or are engaged by, an intervention	*Patient views of the intervention* Is falls prevention a priority for the patient? Previous trial experience	Whether patients felt the intervention would be of benefit Previous experiences of orthotics or exercises Whether reducing risk of falls was a priority for patients Previous and current experience of taking part in research and the trial	Questions on participant baseline questionnaire on participant beliefs and strengths of beliefs of the effectiveness of the intervention	Quantitative description of variation in participant beliefs regarding the effectiveness of the intervention and associated characteristics. Subgroup analysis of trial results according to beliefs and strengths of beliefs of the effectiveness of the intervention at baseline
*Comprehensiveness of intervention description/strategies to facilitate implementation* *How specific is the intervention?* *Intervention complexity* When aiming to evaluate implementation fidelity, what are the specific strategies put in place to optimise the level of fidelity achieved, e.g. provision of manuals, training and incentives?	Practical issues with adhering to and delivering the intervention	Whether podiatrists felt they had received sufficient training and support throughout the trial DVDs and booklets helped podiatrists and patients to deliver and adhere to exercises. Additional follow-up visits and more time to deliver the intervention were recommended by podiatrists ‘Information overload’	Quantitative assessment of adequacy of training, complexity of intervention and confidence in delivering intervention	Quantitative description of variation in perceived ability to deliver intervention and association with delivery as intended
*Quality of delivery* The way that those responsible for delivering the intervention deliver it	Practical issues with adhering to and delivering the intervention	Training and support provided to podiatrists regarding intervention delivery	Qualitative observations to produce quality score for each ‘therapist’	Sensitivity analyses treating fidelity as a measure of compliance Exploring jointly the impact of practitioner fidelity alongside patient compliance within a non-compliance framework
*Program differentiation/Context* Identifying unique features of different components of programs and identifying which elements are essential	How does the REFORM intervention compare to routine practice	Perceived similarities and differences between the trial intervention and routine practice Concerns regarding the time and cost of delivering the intervention	Qualitative work to identify features of complex intervention most likely to be incorporated into routine practice	Descriptively present outcomes by intervention components delivered

A number of frameworks and valid and reliable scales for conceptualising and measuring fidelity exist [5, 6]. However, there is no consensus regarding the best way to measure fidelity or indeed on the ways in which the components of fidelity proposed within existing frameworks impact on specific trial results and how we take this into account in the interpretation of trial findings. This has led to fidelity being overlooked in the past, particularly when designing pragmatic trials.

In the present study, we followed updated Medical Research Council (MRC) guidance [14] and assessed fidelity through a qualitative process evaluation, drawing upon the updated version of Caroll et al.’s conceptual framework [7] to aid how we defined fidelity and analysed and interpreted our findings. Table 3 illustrates how the findings of our process evaluation correspond to nine elements of intervention fidelity as identified by Hasson (some of which have been collapsed down for ease of presentation) [7]. Mapping our findings onto the conceptual framework demonstrates how this can be used to interpret findings within a specific trial, but also enabled us to identify where it would have been useful to collect additional quantitative material and how this could be used to undertake a mixed method analysis to explore the impact of fidelity on trial findings.

For example, in our process evaluation the difference between the REFORM trial intervention and routine practice was considered a key factor influencing the fidelity of the intervention in various ways. For instance, if an intervention which is being introduced as part of a trial is similar to what is routinely prescribed, there may be less resistance and a greater ability for staff to deliver the intervention as intended without adaptation. Equally, this may help implementation in the long term as if an intervention is significantly different from what is routinely prescribed, whether that be in terms of its content or the time and cost of delivery, then there may be barriers at an organisational level, even if the trial shows the intervention improves outcomes. Given that there are a number of ways in which routine practice could influence intervention fidelity, we propose that future trials explore through qualitative process evaluation what the key similarities and differences between the intervention being tested and current routine practice are. From this, it can be hypothesised which elements of a complex intervention are most likely to be used by service providers outside of the context of a trial. Through quantitative recording of whether each component of a complex intervention is delivered, compliance analysis could be conducted to explore whether delivery of the most contextually pragmatic elements of the intervention would be sufficient to see a positive impact on the primary outcome (Table 3).

When considering existing fidelity literature more broadly, one of the shortfalls has been that existing frameworks imply there is a concrete list of factors that affect fidelity, which can be uniformly applied across all trials. This does not allow for flexibility in fidelity measurement or the individual circumstances of each trial to be considered. For instance, the extent that recruitment is a moderator of trial outcomes will vary and, indeed, issues concerning recruitment were perceived to affect the REFORM intervention to a minimal extent. It is therefore not our intention to promote researchers treating the revised version of Carroll et al.’s conceptual framework as a definitive list of factors that will affect trial implementation and outcomes to the same extent. Instead, we wish to encourage flexible use of the framework, with researchers tailoring it to meet the needs of their specific trial and process evaluation. What this means when incorporating fidelity measurement into trial design and evaluation is ensuring that all nine components of fidelity are considered, but acknowledging that certain components will be more relevant to your trial than others.

The limitations of subgroup analyses are well established, including false positives due to multiple comparisons and false negatives due to inadequate power; therefore, we would only suggest careful selection of credible sub-group analysis, as defined by Burke et al. [15]. These include: an assessment of the prior probability for a subgroup effect being present (at least 20% and preferably > 50%); restriction to only one to two primary categorical subgroup analyses; and a priori justification. Another approach would be to undertake sensitivity analyses by treating fidelity as a measure of compliance and to explore jointly the impact of practitioner fidelity alongside patient compliance within a non-compliance framework.

### Strengths and limitations

The present study highlights the usefulness of using qualitative process evaluations to explore the fidelity of complex interventions. The qualitative approach allowed for an in-depth exploration of fidelity to be undertaken, which would not have been possible using other methods such as surveys. Our sample included trial participants and podiatrists, which enabled fidelity to be explored from the perspectives of those who received and delivered the intervention. The main limitation of the present study is that there was no objective measure of fidelity to compare perceptions to, which meant it was not possible to explore the extent by which fidelity affected the trial’s outcomes. While some of the issues identified may be considered unique to the REFORM trial and study population, their application to Caroll et al.’s framework [5] shows that our findings may prove useful to those wishing to consider fidelity when designing future pragmatic trials of complex interventions.

## Conclusion

The updated MRC guidance recognises that intervention fidelity is an under-evaluated issue and state that process evaluations ‘can be used to assess fidelity and quality of implementation, clarify causal mechanisms and identify contextual factors associated with variation in outcomes’ [14]. From the exemplar we have described, we are proposing that qualitative interviews that may be part of a wider process evaluation can be used to generate hypotheses about the key potential moderators for fidelity that are specific to that trial. The hypotheses generated in the process evaluation could then be tested quantitatively in the main trial using sensitivity analysis, drawing on the quantitative measurement of fidelity to give a more complete understanding of trial findings.

## Supplementary information

**Additional file 1.** Topic guide for use with podiatrists. Topic guide used for semi-structured interviews with podiatrists.

**Additional file 2.** Topic guide for use with trial participants. Topic guide used for semi-structured interviews with trial participants.

## Data Availability

The dataset that we have acquired will not be available as our ethical approval does not permit the sharing of the entire dataset that we have acquired.

## Abbreviations

GP: General practitioner
HTA: Health Technology Assessment
MRC: Medical Research Council
NIHR: National Institute for Health Research
NHS: National Health Service
RCT: Randomised controlled trial
REFORM: Reducing Falls with Orthoses and a Multifaceted podiatry intervention
UK: United Kingdom
