# Supplementary material for: Qualitative research to inform hypothesis testing for fidelity-based sub-group analysis in clinical trials: lessons learnt from the process evaluation of a multifaceted podiatry intervention for falls prevention
Source: Trials. 2020 Apr 21;21:348. doi: 10.1186/s13063-020-04274-6 (PMC7171824; doi:10.1186/s13063-020-04274-6)
Supplement: Supplementary file 1 — Additional file 1. Topic guide for use with podiatrists. Topic guide used for semi-structured interviews with podiatrists. [file 13063_2020_4274_MOESM1_ESM.pdf]

Interviews with podiatrists as part of the REFORM study

Invitation telephone call procedure to arrange interview.

1. The qualitative researcher will introduce themselves to the podiatrist as part of the REFORM research team.
2. The researcher will ask the podiatrist if they received the invitation letter.
3. The researcher will explain the reason for calling ie to see if the podiatrist would like to take part in the interview study.
4. The researcher will answer questions and/or explain the study.
5. The researcher will determine if the podiatrist would like to take part in the study.
6. If the podiatrist is willing to take part the researcher will thank them and arrange a convenient date and time. If the podiatrist is not willing to take part, the researcher will thank them for their time.

Interview topic guide for REFORM podiatrists
Approximately 10 podiatrists will be interviewed.

This topic guide summarises the main areas to be explored in each interview about podiatry interventions to improve balance and to reduce the number of falls patients over the age of 70 experience. As with any qualitative interviews, these headings are intended as a starting point to ensure the primary issues are covered, whilst allowing flexibility for new issues to emerge. Preliminary analysis of data from earlier interviews will shape the topics covered in later interviews.

Introduction

- The researcher introduces themselves
- The researcher explains the background of the study
- The researcher should emphasise confidentiality, remind the podiatrist that the interview will be tape recorded and that they can stop the interview at any time if they wish
- The researcher should remind the podiatrist that the information from the research will be written up as a report for the HTA and other reports
- Any questions about the study or interview before we start?

Views on delivering the intervention

- What was your overall impression of the intervention?
- Can you tell me what you liked and disliked about the multifaceted intervention?
- Has there been anything which has hindered your delivery of the intervention? Please describe these and how they could be overcome.
- Have you needed to withdraw a participant from the intervention? If so, why?
- What were your views on the exercise DVD?

THE UNIVERSITY *of York*

- Can you tell me how you see your role in improving balance and reducing the number of falls in elderly adults?

Participating in the study

- Views on their experience of being involved in the REFORM study
- Views about value of the trial

Training

- What did you think about the training you received on how to deliver the multifaceted intervention?
- How could the training sessions have been improved?

Any other issues

- Any other issues or questions the participant would like to raise
- Clarify what happens next in terms of the REFORM study
- Thank them for their time
