# Supplementary material for: Qualitative research to inform hypothesis testing for fidelity-based sub-group analysis in clinical trials: lessons learnt from the process evaluation of a multifaceted podiatry intervention for falls prevention
Source: Trials. 2020 Apr 21;21:348. doi: 10.1186/s13063-020-04274-6 (PMC7171824; doi:10.1186/s13063-020-04274-6)
Supplement: Supplementary file 2 — Additional file 2. Topic guide for use with trial participants. Topic guide used for semi-structured interviews with trial participants. [file 13063_2020_4274_MOESM2_ESM.pdf]

Interviews with participants as part of the REFORM study

Invitation telephone call procedure to arrange interview.

1. The qualitative researcher will introduce themselves to the participant as part of the REFORM research team.
2. The researcher will ask the participant if they received the invitation letter.
3. The researcher will explain the reason for calling ie to see if the participant would like to take part in the interview study.
4. The researcher will answer questions and/or explain the study.
5. The researcher will determine if the participant would like to take part in the study.
6. If the participant is willing to take part the researcher will thank them and arrange a convenient date and time. If the participant is not willing to take part, the researcher will thank them for their time.

Interview topic guide for REFORM participants
Approximately five participants will be interviewed.

This topic guide summarises the main areas to be explored in each interview about podiatry interventions to improve balance and to reduce the number of falls patients over the age of 70 experience. As with any qualitative interviews, these headings are intended as a starting point to ensure the primary issues are covered, whilst allowing flexibility for new issues to emerge. Preliminary analysis of data from earlier interviews will shape the topics covered in later interviews.

Introduction

- The researcher introduces themselves
- The researcher explains the background of the study
- The researcher should emphasise confidentiality, remind the participant that the interview will be tape recorded and that they can stop the interview at any time if they wish
- The researcher should remind the participant that the information from the research will be written up as a report for the HTA and other reports
- Any questions about the study or interview before we start?

Understanding improving balance and reducing the number of falls

- General background information – family circumstances, general wellbeing and their personal history of balance problems and falls. The impact of any balance problems or falls.

- Ask about the study participant's understanding of improving balance and reducing the number of falls, where they have gained this information from (family, friends, professionals, media) how much of a problem they perceive this to be
- Ask the participant about their thoughts on the relationship between improving balance and reducing falls on any current health problems they are currently experiencing
- Explore the extent to which improving balance and reducing falls is an important issue to the participant. Eg are they steady on their feet, if they've had any fractures, worry about falling, if they would undertake any steps to reduce the possibility of them falling
- Ask the participant about their understanding of 'risk factors' what kind of things do they think might cause older adults, friends or themselves to have poor balance and fall. If they consider themselves at risk of falling
- Ask about their views and how they manage improving balance and preventing falls or why not if this is not done
- Ask about any perceived improvements in balance/falls since the start of the study

For participants allocated to the intervention group

- What did you think about the orthosis?
- Did you think they were a good idea?
- How often did you wear the orthotics?
- Did you have any problems with wearing the orthotics?
- Did you think the orthotics would help?
- Do you feel more confident when wearing the orthotics?
- Does wearing the orthotic make you worry less about falling?
- What did you think about the foot and ankle exercises?
- Did you find it easy to undertake them three times a week?
- Did you think the exercises would help?
- Did you have any problems undertaking the exercises?
- Did you manage to fill in the exercise diary?
- Did you receive any footwear advice?
- Were you given a voucher to buy new shoes? If 'yes' did you buy new shoes? If not, why not.

For participants allocated to the control group

- *Whether they have introduced any measures to address their balance problems, did they follow any advice from the falls prevention leaflet?*

Participating in the study

- Explore the participant's views on their experience of being involved in the REFORM study eg was it something that they really wanted to do? Have they ever taken part in a study before? Would they take part in a research study again?
- Views on being randomised to the intervention group eg experiences of randomisation process and understanding of this, feeling about completing questionnaires for study
- Views about value of the trial
- How did you feel about filling in the falls calendar? Did you manage to fill it in? Did you have any problems? What did you think of the layout? Could it have been improved in any way?
- What did you think about the other questionnaires we asked you to complete? How did you find these? Were they easy to understand? What was the layout like? Could they be improved in any way?
- What did you think about the falls prevention leaflet we sent out?

Any other issues

- Any other issues or questions the participant would like to raise?
- The researcher will clarify what happens next in terms of the REFORM study
- The researcher will thank the participant for their time.
